# Nanoalginates via Inverse-Micelle Synthesis: Doxorubicin-Encapsulation and Breast Cancer Cytotoxicity

**DOI:** 10.1186/s11671-018-2748-2

**Published:** 2018-11-03

**Authors:** Justin G. Rosch, Anna L. Brown, Allison N. DuRoss, Erin L. DuRoss, Gaurav Sahay, Conroy Sun

**Affiliations:** 1 Department of Pharmaceutical Sciences, College of Pharmacy, Oregon State University, Portland, OR 97201 USA; 2 Department of Biomedical Engineering, School of Medicine, Oregon Health & Science University, Portland, OR 97201 USA; 3 Department of Radiation Medicine, School of Medicine, Oregon Health & Science University, Portland, OR 97239 USA

**Keywords:** Alginate, Nanoparticles, Doxorubicin, Breast cancer, Cell viability

## Abstract

Crosslinked-biopolymer nanoparticles provide a convenient platform for therapeutic encapsulation and delivery. Here, we present a robust inverse-micelle process to load water-soluble drugs into a calcium-crosslinked alginate matrix. The utility of the resulting nanoalginate (NALG) carriers was assessed by a doxorubicin (DOX) formulation (NALG-DOX) and evaluating its potency on breast cancer cells (4T1). This facile synthesis process produced doxorubicin-containing particles of ~ 83 nm by hydrodynamic size and zeta potential ~ 7.2 mV. The cyclohexane/dodecylamine microemulsion yielded uniform and spherical nanoparticles as observed by electron microscopy. The uptake of the drug from the NALG-DOX formulation in 4T1 cells was observed by fluorescence microscopy employing doxorubicin’s inherent fluorescence. Therapeutic efficacy of the NALG-DOX against 4T1 cells was demonstrated qualitatively through a LIVE/DEAD fluorescence assay and quantitatively via cell viability assay (Alamar Blue). In addition, IC_50_ values were determined, with encapsulated doxorubicin having a slightly higher value. No toxicity of the empty NALG carrier was observed. Overall, these results demonstrate the utility of this synthesis process for encapsulation of hydrophilic therapeutics and NALG to function as a drug carrier.

## Background

Encapsulation of therapeutic payloads offers many advantages over systemic administration of free drugs into the bloodstream. Increased circulation time [1–3], shielding from plasma proteins [4], and lower systemic toxicity [5, 6] achieved by nanocarrier delivery may significantly improve therapeutic efficacy. In addition, the enhanced permeability and retention of solid tumors can be leveraged for passive targeting if the therapeutics are encapsulated in carriers of the appropriate size or type [7–9]. Numerous therapeutics have been incorporated into nanocarriers including doxorubicin (DOX) [6, 10–12], cisplatin [13, 14], and paclitaxel [15–17]. Also, many carrier technologies, such as liposomes [18–21], polymer-based carriers [15, 22–25], and lipid nanoparticles [26–29], have begun making an impact on clinical outcomes. However, translation of novel formulations is often hindered by challenges in preparation and processing of these compounds. Here, we present a simple, reliable, and robust method of producing biocompatible alginate nanocarriers capable of encapsulating hydrophilic chemotherapeutic agents.

Biopolymers have been used to encapsulate therapeutics in part due to their ease of use and their biocompatible nature [30]. Polymers used include alginate [31, 32], heparin [33, 34], chitosan [35, 36], and carrageenan [37, 38], among others. Alginate, a naturally derived polymer, is composed of varied amounts of β-d-mannuronate and α-l-guluronate residues linked by 1,4-glycosidic linkages [39] (M and G blocks, respectively). Alginate can be crosslinked via addition of multivalent cations, of which calcium is commonly used [40–42]. The presence of calcium can lead to the formation of larger packed structures between linked G blocks, referred to as “egg-box” structures [43]. Crosslinking of the alginate strands in solution creates a hydrogel. Other polymers, such as chitosan [43–45], influence structural properties of the particle. The hydroxyl and carboxylic acid groups on the alginate chains confer a negative overall charge to assembled structures of the polymer [14, 46]. By linking the carboxylic acids and forming a three-dimensional matrix, therapeutics can be entrapped.

Doxorubicin is a widely used chemotherapeutic employed in the treatment of various cancers [47]. Doxorubicin’s primary mechanism of action involves association with replication-associated enzymes, which allows intercalation into DNA strands. It is also capable of direct insertion into the DNA strand. The result is disruption of the replication process, which prevents cell proliferation, and leads to apoptosis [48]. In addition to this mechanism, doxorubicin is associated with reactive oxygen species generation in the cell [48, 49]. Doxorubicin is highly effective [6, 50], but has serious toxic effects involving multiple organs, including the heart [51, 52], brain [53], liver [47], and kidneys [54]. Encapsulation can lead to reduced systemic toxicity, with liposomal Doxil becoming a well-known success [7].

Alginate is a low-cost, biocompatible, biodegradable, and easily sourced substance, and is generally considered non-immunogenic [55, 56]. Hydrogel nanoparticles formed by crosslinked alginate have been used for the encapsulation of various therapeutics [56]. These processes vary from surfactant-driven formation of inverse micelles [39] to mechanical stimuli or temperature-induced formation of particles [41]. We present a simple and robust means of producing relatively monodisperse alginate nanocarriers. This synthesis process is carried out at room temperature (unlike as presented by Machado et al.) and can be performed in just a few hours. The inverse micelle process incorporates aqueous-soluble therapeutics into the alginate matrix, without the need for chemical modification. Dynamic light scattering (DLS) measurements of the alginate nanoparticles showed uniform distributions of particles ~ 80–90 nm. Electron microscopy confirmed the sizes and the rough spherical morphology of the particles. Doxorubicin encapsulated in the alginate matrix of the nanoparticles shows distinct in vitro efficacy relative to free doxorubicin, indicating that future studies could investigate the efficacy of the therapeutics in vivo.

## Methods

### Materials

Sodium alginate, calcium chloride dihydrate, cyclohexane (99.9%), and dodecylamine (98%) were purchased from Sigma-Aldrich (St. Louis, MO). Doxorubicin hydrochloride was purchased from MedChem Express (Monmouth Junction, NJ). These reagents were used as is, with dissolution of sodium alginate and the therapeutics in water as indicated. All water was 18 MΩ filtered water provided from a Milli-Q source.

### Preparation of Nanoalginates

Sodium alginate was dissolved in a glass vial at 15 mg/mL in water and allowed to mix via a stir bar for at least 30 min prior to use. The DOX was dissolved in this aqueous phase if it was to be incorporated into the nanocarrier. The alginate solution was checked for complete dissolution of the alginate powder prior to use in synthesis. Without the introduction of a multivalent cation, the aqueous alginate phase remains homogeneous for weeks. Eight milliliters of cyclohexane was pipetted into a vial. Dodecylamine, a solid at room temperature, was heated under warm water for a few minutes, until some of the solid had been converted to liquid form. Then, 80 μL of dodecylamine was then pipetted into the cyclohexane. A stir bar was added to the glass vial, and the mixture was then stirred at 125 rpm. After 5 min of mixing, the organic phase was considered well-mixed and ready for addition of the aqueous phase. Twenty microliters of the aqueous alginate phase was added to the cyclohexane/dodecylamine organic phase. The stirring rate was increased to 1200 rpm. Mixing occurred under constant stirring for 20 min. Thirty microliters of 50 mM calcium chloride solution was then added to the mixture. After 25 min of mixing/gelation of the particles, 2 mL of water was added to the mixture, creating an aqueous layer underneath the organic layer. The nanoparticles separated into the aqueous phase, and a 1 mL pipette was used to remove the aqueous layer.

### Purification of NALG

The aqueous layer was spun in a 100 kDa centrifugal filter unit (Pall) for 10 min at 3200×*g* in a centrifuge to remove large aggregates, and the permeate was transferred to a 10 kDa unit (Millipore). The solution was then spun for 5 min at the same speed to filter out small impurities. Water was added and spun for 5 min to wash the retentate. The final volume of 1 mL was collected, and characterization was performed on this product.

### Characterization of the NALG

The filtered solution of nanoparticles was transferred to a cuvette (Malvern Instruments, DTS 1070 zeta cell) for dynamic light scattering measurement. The size distribution was determined using a Malvern Zetasizer ZSP (Malvern Instruments, UK). Size measurements were performed using a 633 nm wavelength laser at 25 °C with a detection method of 173° backscatter angle. Zeta potential measurements for the alginate nanoparticles were performed in the same cuvette immediately following the size measurement. Measurements for size and zeta potential were performed in triplicate, and results presented indicate the mean ± standard deviation of the three trials. Transmission electron microscopy (TEM) was used to confirm size of the nanoparticles and investigate their morphology. TEM images were acquired using a Technai F-20 transmission electron microscope operating at 4200 eV. Preparation of the TEM grids involved dropwise deposition of 10 μL of post-filtered nanoparticle solution onto the copper surface of the formavar/carbon backed TEM grid (Ted Pella). Wet TEM grids were placed covered in a desiccator overnight prior to imaging to ensure proper drying.

Doxorubicin has intrinsic fluorescence, which can be used to compare the fluorescence of the doxorubicin alginate nanoparticles (NALG-DOX) to a standard curve of DOX to estimate its concentration. We used excitation/emission wavelengths of 470/550 nm. Release of doxorubicin from NALG-DOX solutions was determined by dialyzing the final filtered nanoparticle solution into phosphate-buffered saline (PBS) at pH 7.4 or citrate buffer at pH 5.5. Two sets of five batches of NALG-DOX were prepared as described previously, with an initial DOX concentration of 1.25 mg/mL in the aqueous alginate phase. Eighteen Slide-A-Lyzer MINI Dialysis devices (ThermoFisher) with an inner volume of 100 μL and a molecular weight cutoff of 2 kDa were prepared by adding 100 μL of NALG-DOX to each device. Each device was placed in a scintillation vial containing 20 mL of buffer. A stir bar was added to each buffer-containing vial, and all vials were placed on a stir plate for gentle agitation for the duration of the experiment. The vials were covered to reduce loss of the buffer to evaporation, and to protect from light. At each time point (1, 2, 4, 24, 48, 72 h), three devices were removed, 100 μL of the sample was removed from each device and placed in a separate well, and a fluorescence measurement was made on a Tecan Infinite M200 Pro microplate reader (Tecan Trading AG) at 470/550 nm. The fluorescence measurements were compared to measurements from a standard curve and initial aliquots taken before the experiment to determine the concentration and the amount of loss of DOX to the buffer.

### Cell Culture and Dosing

Murine breast cancer cells, Bioware Ultra Green Cell Line 4T1 luciferase/green fluorescent protein (4T1-luc2-GFP, mouse adenocarcinoma) and no reporter 4T1 cells were obtained from PerkinElmer (Waltham, MA). These cells were maintained with RPMI 1640 medium supplemented with 10% fetal bovine serum (FBS) and 1% penicillin/streptomycin in a 5% CO_2_ incubator operating at 37 °C. The 4T1 cells were seeded at 4000 cells/well in a 96-well black walled, clear bottom plate. Twenty-four hours after seeding in the wells, the initial media was aspirated, and the drug/media formulation was introduced.

### Fluorescence Imaging

At 48 h, the media was aspirated, the cells were washed three times with PBS, and formalin was added to the wells. After 30 min of room temperature incubation, the cells were again washed three times with PBS, and two drops of NucBlue fixed cell ReadyProbes reagent (ThermoFisher) per milliliter of media was added to the wells. After 1 h of room temperature incubation, the cells were again washed three times with PBS. Then, 100 μL of PBS was added to the fixed cells, and the plates were transferred to an EVOS FL Auto Cell Imaging System (ThermoFisher). A × 20 objective was used to image parts of certain wells in each plate. An image of each fluorescence channel was recorded separately; a blue image for 4′,6-diamidino-2-phenylindole (DAPI), a red image for DOX (using RFP channel to capture intrinsic fluorescence), and a green image for GFP. The 4T1-luc2-GFP cells express green fluorescent protein, so no additional staining was required for the cells to be visible in the green channel. The DOX treatment “stains” the cells, and no further reagent is needed to visualize it in the red channel. The images were brightness/contrast adjusted in ImageJ.

### Live/Dead Cell Viability Assay

Qualitative assessment of the viability of 4T1 cells after coincubation with NALG, DOX, and NALG-DOX was evaluated using a LIVE/DEAD cell viability assay (ThermoFisher). At 72 h after introduction of the drug laden media, the media was aspirated, and the cells were washed three times with PBS. The LIVE/DEAD assay was performed by adding two drops from each dropper of NucBlue Live Reagent and NucGreen Dead Reagent (ThermoFisher) per milliliter of media. Three more washes with PBS were performed after 30 min of incubation, and formalin was added to the wells to fix the cells. Three more washes with PBS followed the formalin, and a final 100 μL amount of PBS was added to each well. Similar imaging was performed as stated, but the green channel now indicated the presence of dead cells, as the compromised cell membranes allowed the green reagent to enter the cell.

### Live/Dead Cell Viability Assay

Quantitative assessment of the viability of 4T1 cells after coincubation with NALG, DOX, and NALG-DOX was evaluated using Alamar Blue cell viability assay (ThermoFisher). At 72 h after introduction of the drug laden media, the media was aspirated. The Alamar Blue assay was performed by adding 10 μL of Alamar Blue reagent to each well with 100 μL of new media and incubated for 1 h at 37 °C. After 1 h, the cell viability was determined for each well using a Tecan Infinite M200 Pro microplate reader (Tecan Trading AG). The excitation/emission wavelengths were set to 560/590, optimal gain settings were determined for the plate by the software, and a bottom read protocol was used to read the fluorescence intensity for each well in the plate. The % cell viability could be determined from the fluorescence intensity readout via the equation:

$$ \%\mathrm{cell}\ \mathrm{viability}=\frac{\mathrm{fluorescence}\ \mathrm{intensity}\ \mathrm{of}\ \mathrm{well}}{\mathrm{fluorescence}\ \mathrm{intensity}\ \mathrm{of}\ \mathrm{no}\ \mathrm{treatment}\ \mathrm{well}}\ast 100\% $$

## Results and Discussions

### Inverse Micelle Emulsion Process

**Fig. 1:**
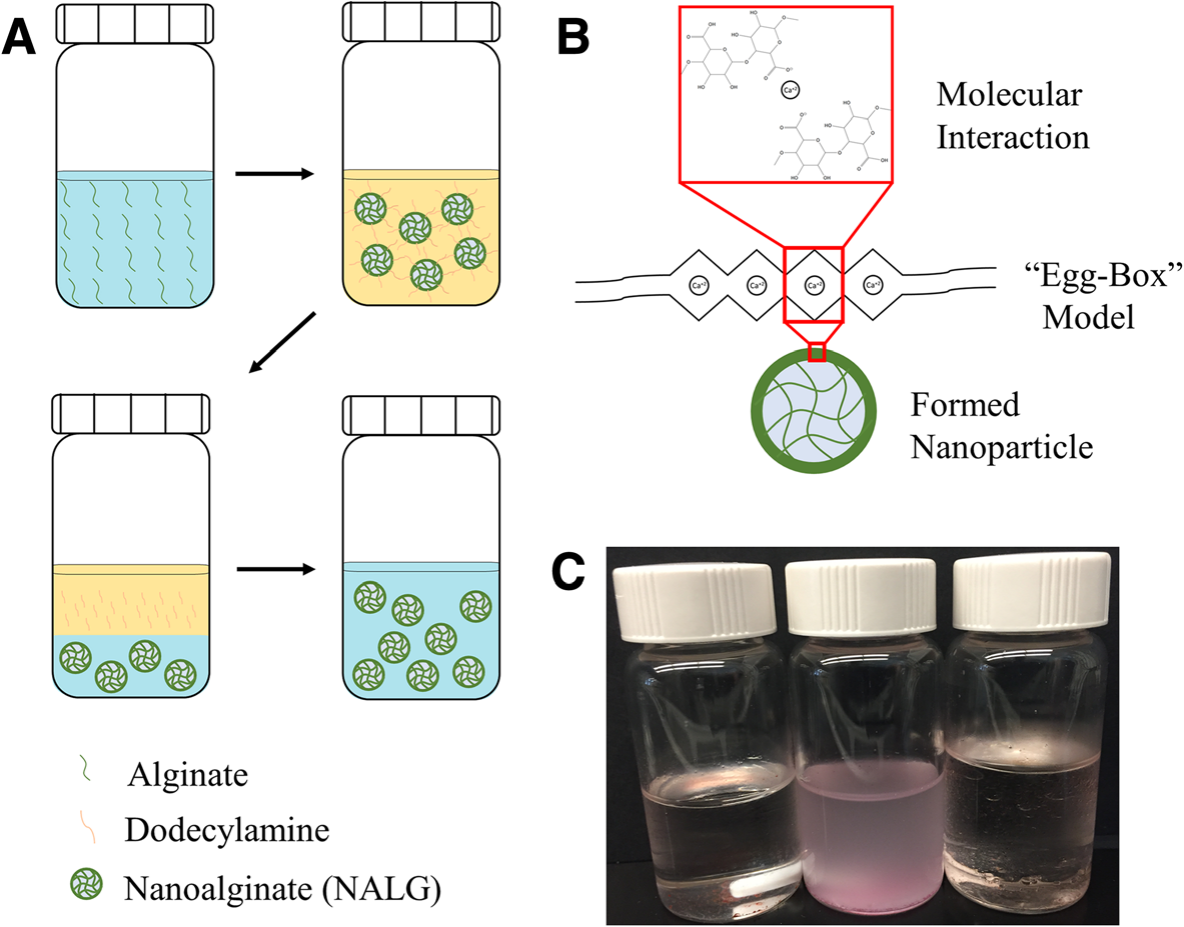
Formulation process for formation of NALG and NALG-DOX. **a** Schematic representation of inverse micelle synthesis process. Aqueous alginate chains are crosslinked in organic bath (top), extracted to an aqueous phase (bottom left), then filtered for purification (bottom right). **b** Molecular interaction of Ca^2+^ crosslinking, leading to formed NALG. **c** Photograph of synthesis at various points, from left to right, emulsion before CaCl_2_ addition, emulsion after CaCl_2_ addition, and separation upon addition of water

In this work, a nanoalginate drug carrier, NALG, was prepared via an inverse micelle emulsion process, as illustrated in Fig. 1. Aqueous alginate was added to a cyclohexane/dodecylamine oil phase, which forms the inverse micelles. The alginate was then crosslinked with addition of calcium chloride (CaCl_2_) solution. After time passed to allow crosslinking of the alginate within the inverse micelles, the NALG were extracted by addition of water. Finally, centrifugal filters removed latent contaminants and helped narrow the size distribution of the nanoparticles. The final product was a transparent aqueous colloidal solution.

The components of the inverse micelle emulsion system were determined based on experimental data and past literature. To narrow the degrees of freedom in the system, we chose cyclohexane for the organic phase. In the optimization of this process, we tested an array of surfactants for their ability of form nanoscale alginate particles indicated by an increase in turbidity of the organic mixture upon introduction of a small amount of aqueous phase while stirring. Dodecylamine was a suitable candidate and was chosen as the surfactant to use moving forward. Water was chosen as the aqueous phase in the experiments presented here.

### Characterization of NALG

**Fig. 2:**
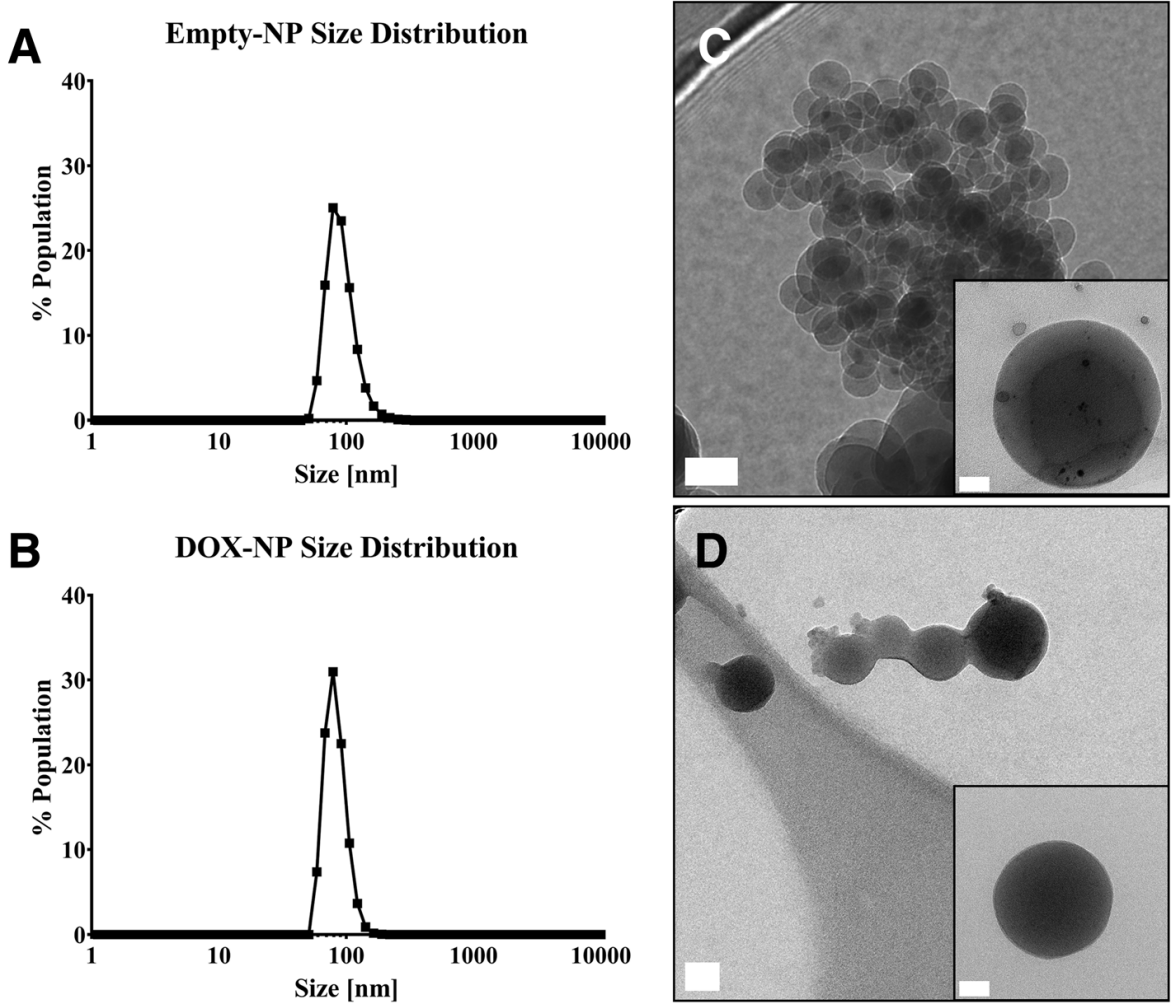
Dynamic light scattering distributions of **a** NALG and **b** NALG-DOX show monodisperse distributions of alginate nanoparticles. Transmission electron microscopy images of **c** NALG and **d** NALG-DOX show spherical morphology of particles. Scale bars indicate 50 nm in larger picture, and 20 nm in inset

In Fig. 2a, b, the distribution of nanoparticle hydrodynamic sizes for NALG and NALG-DOX are shown. The nanoparticles, whether empty or therapeutically loaded, showed similar peaks centered around 90 nm. The particle diameters for NALG and NALG-DOX, respectively, were 92.2 ± 4.2 nm and 82.8 ± 3.6 nm. The polydispersity index (PDI) for the particles were 0.320 ± 0.063 and 0.204 ± 0.044, and the ζ-potentials were − 15.0 ± 0.8 mV and 7.2 ± 4.6 mV.

The sizes of the particles were consistent upon incorporation of the DOX into the alginate matrix. The ζ-potential was negative for the empty NALG, which was consistent with other alginate nanoparticles linked by calcium [39]. The doxorubicin-loaded particles possessed a positive ζ-potential. This could be due in part to an excess of positively charged calcium ions at the surface of the particle, which may help impart the positive charge on the nanoparticle. Also, the primary amine present on DOX molecules can electrostatically interact with the free carboxylic acid groups on the alginate, reducing the negative charges available on the surface of the particle.

Figure 2c, d shows transmission electron microscopy images of calcium-crosslinked NALG and NALG-DOX. Both types of nanoparticles displayed spherical morphology. The general distribution of nanoparticles appeared to be similar in size to the DLS distribution. The concentration of the final NALG-DOX solution was evaluated using the intrinsic fluorescence of the doxorubicin molecule. The volume of the filtered NALG-DOX product was divided up into triplicate sets of dialysis devices placed into PBS-filled scintillation vials and DOX was allowed various times to release from the NALG-DOX. Initially, the concentration of DOX in the NALG-DOX solution was ~  4 μg/mL, indicating that the encapsulation efficiency, or ratio of DOX in NALG-DOX to initial DOX added to formulation, for the NALG-DOX is ~ 7%. While this encapsulation efficiency is considered low compared to similar particles [57], this is likely due to the initial DOX present in the aqueous phase. We did not iterate on the formulation by lowering the amount of DOX present in the initial aqueous phase and hypothesize that the efficiency could be improved by lowering the amount of DOX in the initial formulation. The unencapsulated DOX is likely passing through the 3 kDa filter washes at the end of the synthesis. With the availability and relative low cost of doxorubicin, we opted to “saturate” the formulation with DOX, knowing that large amounts of the leftover DOX would likely pass through unencapsulated. Increasing the encapsulation efficiency could improve this particle platform in future work.

**Fig. 3:**
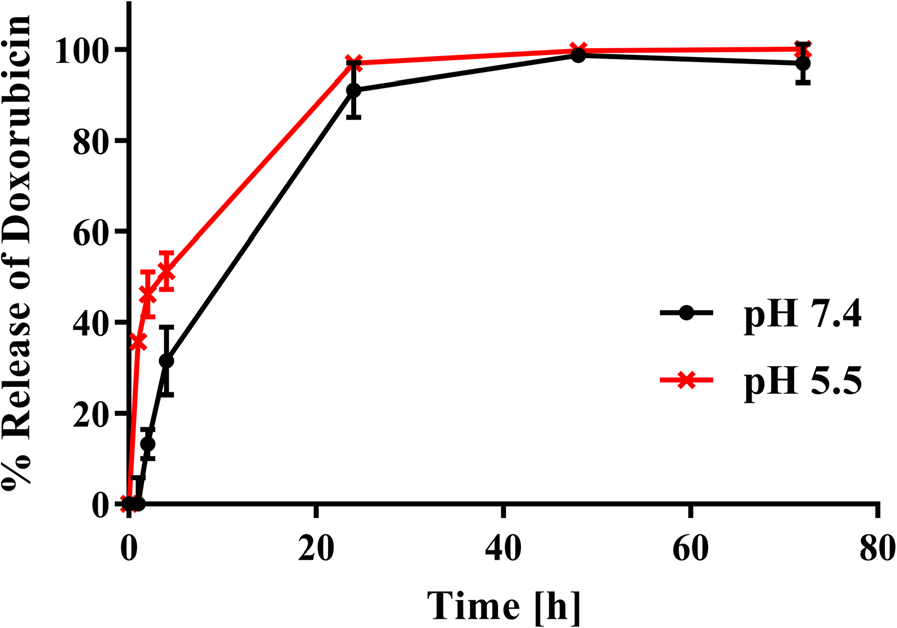
Release of doxorubicin from nanoparticle solution shows controlled release over first 24 h in PBS, pH 7.4. Faster release of doxorubicin occurs at pH 5.5. Each point represents mean ± standard deviation of *n* = 3 measurements

The release curve for the NALG-DOX at pH 5.5 and 7.4 can be found in Fig. 3. NALG-DOX retains approximately 70% of the DOX payload over the course of the first 4 h, with 90% leaving the particles by 24 h, at pH 7.4. This release pattern is common for polymeric materials [57, 58], and demonstrates controlled release over the first 24 h when exposed to the PBS reservoir. At a lower pH of 5.5, more similar to the pH the nanoparticles would see during cell uptake in a late endosome [59], the DOX is released at a faster rate. This is desired behavior, as slower release is needed when in general circulation in the blood, but when in the acidic tumor microenvironment, faster release is preferred.

### Cellular Uptake of DOX

**Fig. 4:**
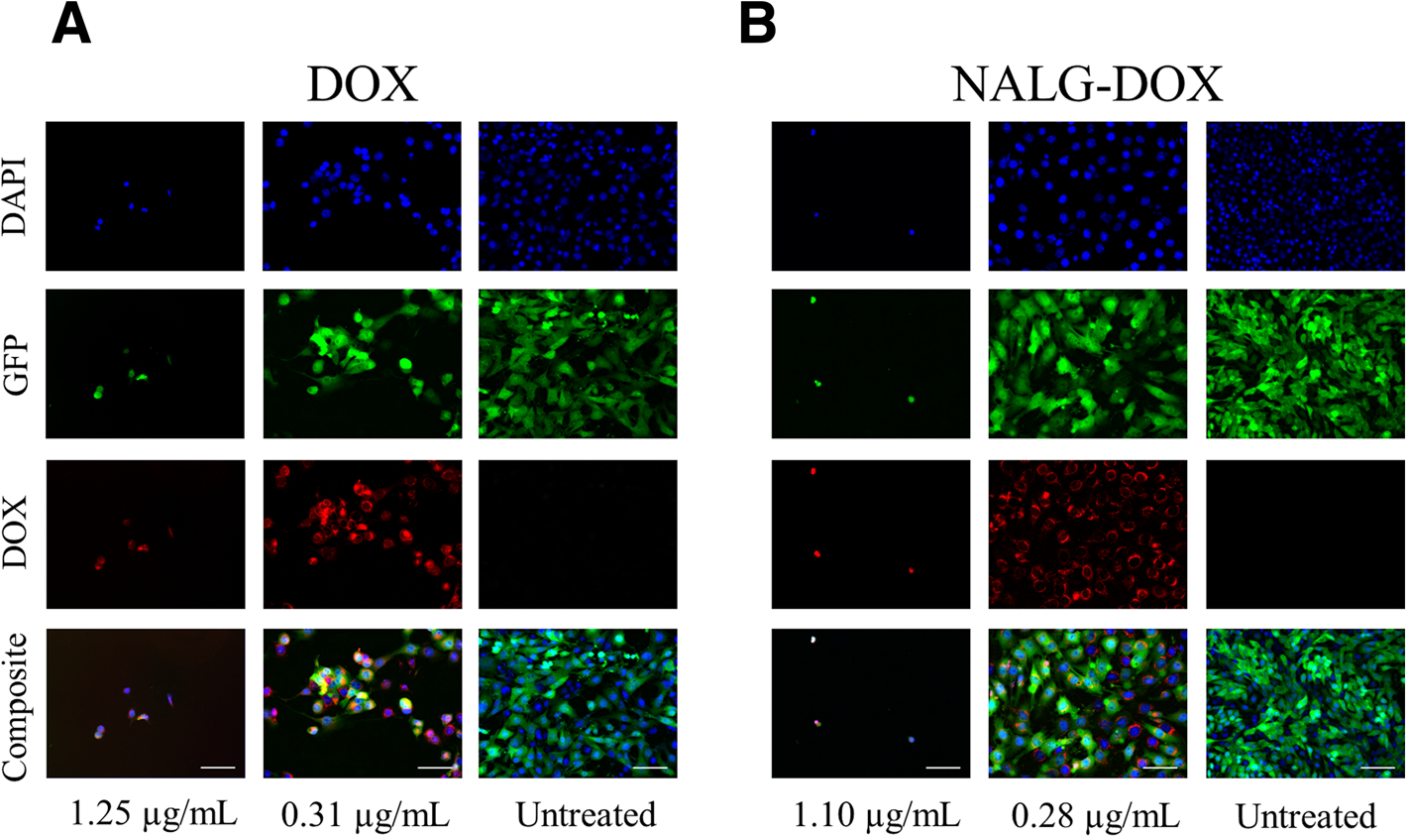
Fluorescence microscopy of images of fixed 4T1-luc2-GFP breast cancer cells treated with doxorubicin (DOX) (**a**) and NALG-DOX (**b**) after 48-h exposure. Scale bars indicate 100 μm. Blue nuclear stain: DAPI; green (transfected cell line): GFP; red (intrinsic molecular fluorescence): DOX

The uptake of NALG-DOX by 4T1 mouse breast cancer cells at 48 h is shown in Fig. 4a, b. Each row indicates a separate color channel, and the bottom row shows the composite image of all color channels. In the left column, a high concentration of DOX or NALG-DOX is used, which is sufficient to eliminate most of the cells. The middle column shows a concentration of 0.31 μg/mL for DOX, and 0.28 μg/mL for the DOX in the NALG-DOX, which shows some cell killing potential, and the right column shows untreated cells. Cells in the center image show doxorubicin (red) around and overlapping with the nucleus (blue) of the cells. There are reduced cell numbers in the first two columns in both panels a and b due to the presence of the DOX, which inhibited cell proliferation in comparison to the untreated wells. Doxorubicin functions through intercalation into the nuclear DNA [12], and colocalization of the doxorubicin and the nucleus could indicate that it is functioning through this mechanism upon uptake. In Fig. 4b, the encapsulated doxorubicin is distinct in the periphery of the nuclear area. The right column shows untreated cells with no significant signal detected in the red channel. Free DOX, while potent, would inflict off target toxicity and have reduced circulation times in vivo. Encapsulation allows for more steady release over increased circulation time, making NALG-DOX potentially better in vivo, and could be explored in future work.

### Cytotoxicity of NALG-DOX

**Fig. 5:**
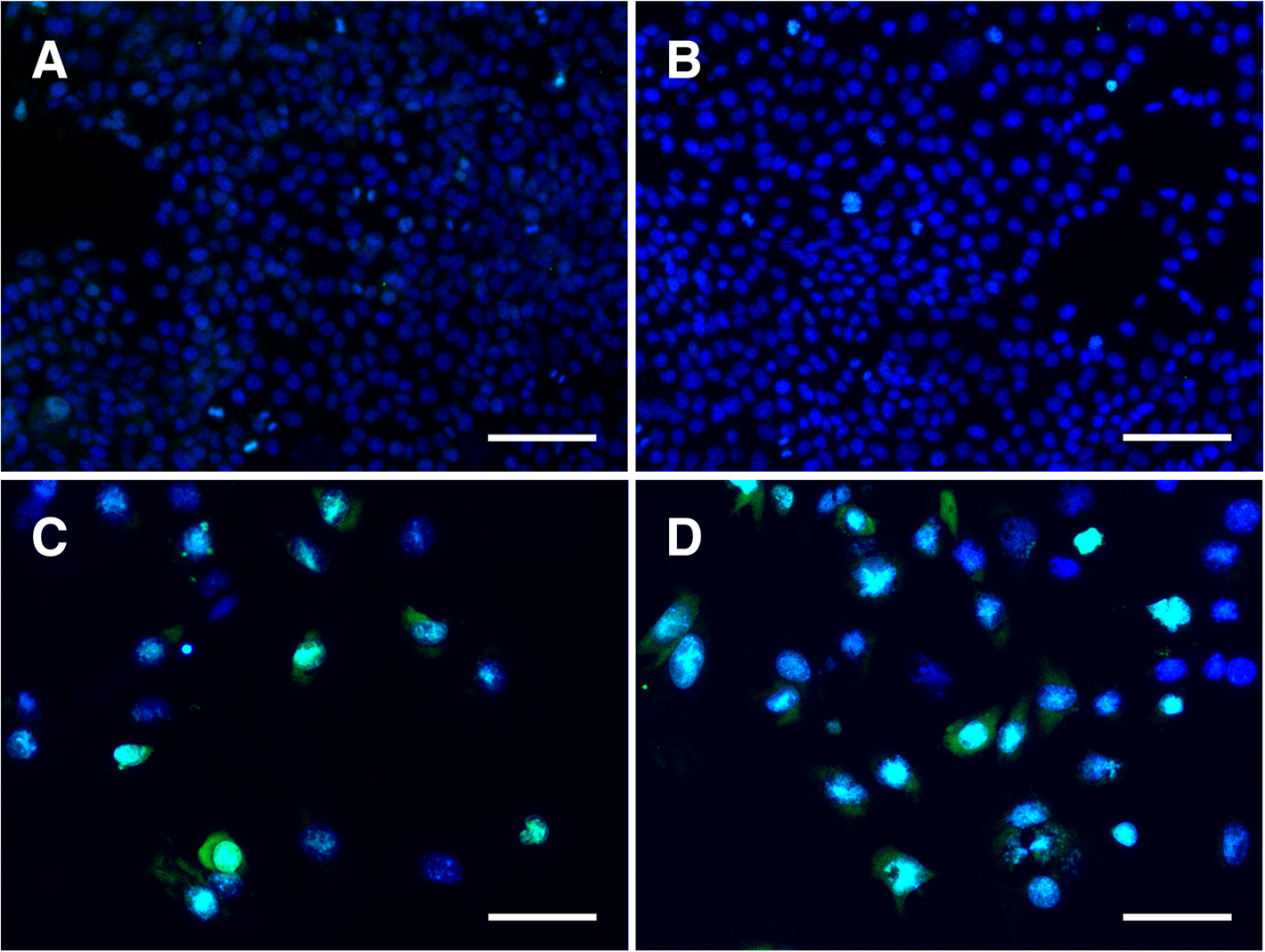
LIVE/DEAD fluorescence assay demonstrates little to no cell death in untreated 4T1 cells (**a**) and in cells treated with NALG (**b**). Green channel signal intensity (dead cell stain) is strong in a large amount of the cells treated with 0.078 μg/mL DOX (**c**) and 0.20 μg/mL NALG-DOX (**d**), indicating cell death. Scale bars indicate 100 μm. Concentrations indicate amount of DOX, either free or in particle. NALG dosing in (**b**) at same particle density as in NALG-DOX

As a basic test for cell killing potential, untreated 4T1 cells and cells treated with NALG-DOX were stained with LIVE/DEAD cell assay (ThermoFisher) and fixed after 72 h of exposure. An example image from an untreated well, a well treated with NALG, a well treated with 0.078 μg/mL DOX, and a well treated with 0.20 μg/mL NALG-DOX, is shown in Fig. 5a–d, respectively. The green overlap shown in Fig. 5c, d indicates that the DOX and NALG-DOX treatment in those wells lead to the death of many of the cells in that well. The cells were not able to proliferate in the same manner as in the untreated and empty nanoparticle-treated wells shown in Fig. 5a, b due to the presence of the therapeutic. The dilution of nanoparticles used for dosing in NALG and NALG-DOX was identical.

**Fig. 6:**
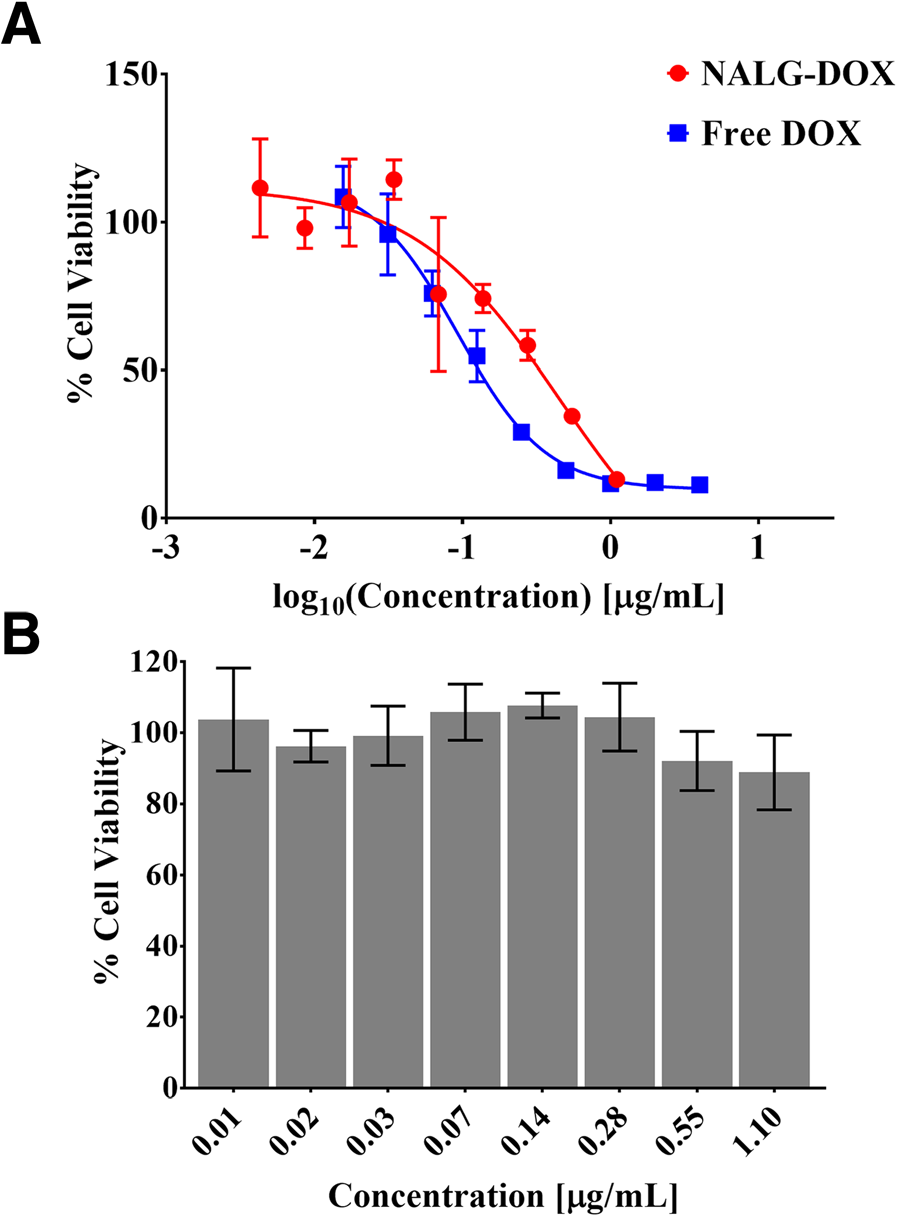
Cell viability of 4T1-luc2-GFP cells after 72-h exposure to NALG-DOX and DOX (**a**) and NALG (**b**) at various concentrations. Data points and error bars indicate mean ± standard deviation from *n* = 3 wells. Concentrations indicate amount of DOX, either free or in particle, in (**a**). In **b**, NALG wells were dosed starting at same particle concentrations as NALG-DOX, indicating little to no cell death from drug carrier alone

Quantitative assessment of cell viability of the 4T1-luc2-GFP cells after 72-h exposure to NALG, free DOX, and NALG-DOX via Alamar Blue (ThermoFisher) assay is shown in Fig. 6. In Fig. 6a, the free doxorubicin-treated cells showed less viable cells across the entire concentration range than the NALG-DOX-treated cells (for equivalent concentration of DOX). This is further displayed by the IC_50_ values, or concentration necessary to show a 50% inhibitory effect, which were 0.093 μg/mL and 0.45 μg/mL, for free DOX and NALG-DOX respectively. The free DOX value is similar to that found at 72 h against 4T1 cells by Du et al. [60]. The IC_50_ values are similar to those found by Eliaz et al. with DOX and liposome-encapsulated DOX at 72 h against B16F10 melanoma cells [61].

A higher concentration of doxorubicin is needed for the NALG-DOX to show a similar effect to the free drug. This should be expected, as the encapsulated therapeutic is more hindered in its transport to its site of effect. The encapsulated doxorubicin still showed cell inhibitory effects, meaning either the drug is still therapeutically active or the nanoparticle itself is toxic to the cells. To test for this, NALG viability was evaluated in a similar manner to NALG-DOX. NALG showed minimal toxicity across all concentrations, as shown in Fig. 6b, indicating that the drug is efficacious.

## Conclusions

We have developed an inverse micelle platform capable of producing spherical alginate nanoparticles that are ~ 90 nm in size. NALG-DOX uptake was examined in 4T1-luc2-GFP breast cancer cells and showed distinct uptake to locations near the nucleus when encapsulated in the alginate carrier. Cell toxicity of the free drug versus the encapsulated drug was compared by examining therapeutic IC_50_ values. Encapsulated doxorubicin showed lower toxicity when compared to its free drug counterpart. These NALG-DOX may be of great interest for drug delivery purposes, as off target effects of the doxorubicin would be reduced in systemic dosing of the encapsulated form. Future formulations will be optimized for slower controlled release profiles and increased encapsulation. This facile process provides an efficient synthetic route that can be completed in just a few hours, allowing further characterization, in vitro, and in vivo experimentation to proceed quickly.

## Abbreviations

4T1-luc2-GFP: 4T1 luciferase/green fluorescent protein
CaCl_2_: Calcium chloride
DAPI: 4′,6-Diamidino-2-phenylindole
DLS: Dynamic light scattering
DMSO: Dimethyl sulfoxide
DOX: Doxorubicin
NALG: Alginate nanoparticles
NALG-DOX: Doxorubicin alginate nanoparticles
PBS: Phosphate-buffered saline
PDI: Polydispersity index
TEM: Transmission electron microscopy
